# Replacement of Soybean Meal With Soybean Cake Reduces Methane Emissions in Dairy Cows and an Assessment of a Face-Mask Technique for Methane Measurement

**DOI:** 10.3389/fvets.2019.00295

**Published:** 2019-09-04

**Authors:** Sylvia Rocha Silveira, Stephanie Amelia Terry, Tamara Elaine Biffin, Rogério Martins Maurício, Luiz Gustavo Ribeiro Pereira, Alexandre Lima Ferreira, Rafael Sandin Ribeiro, João Paulo Sacramento, Thierry Ribeiro Tomich, Fernanda S. Machado, Mariana. M. Campos, Marco Antonio Sundfeld Gama, Alexandre Vieira Chaves

**Affiliations:** ^1^Bioengineering Department, Universidade Federal de São João del-Rei, São João del-Rei, Brazil; ^2^Faculty of Science, School of Life and Environmental Sciences, University of Sydney, Sydney, NSW, Australia; ^3^Faculty of Science, Sydney School of Veterinary Science, University of Sydney, Sydney, NSW, Australia; ^4^Brazilian Agricultural Research Corporation—Embrapa Dairy Cattle, Juiz de Fora, Brazil

**Keywords:** co-product, dairy cattle, greenhouse gas, climate change, respiration chamber

## Abstract

The objective of this study was to (a) evaluate the effect of replacing soybean meal (SBM) with soybean cake (SBC) on feeding behavior, rumen fermentation, milk production, nutrient digestibility and CH_4_ emissions and (b) investigate whether a face-mask technique could be used to predict daily methane (CH_4_) emissions in dairy cattle. The experiment was conducted as a completely randomized design, with 32 crossbred Holstein × Gyr cows (days in milk (DIM): 112 ± 25.1) randomly assigned to the following treatments (*n* = 8/group) for 75 days: (1) 0% SBC, (2) 6% SBC, (3) 14% SBC, and (4) 23% SBC, in place of SBM on a dry matter (DM) basis. Across the final 4 weeks of the study, CH_4_ production was estimated using the proposed face-mask technique subsequent to a respiration chamber measurement for an evaluation of treatment efficacy and face-mask accuracy. There was no effect of SBM replacement by SBC on intake, feeding or drinking behavior (*P* > 0.21). Total VFA concentration, the individual proportions of VFA and blood metabolites were not altered (*P* > 0.17) by SBC, however there was a tendency for decreased (*P* = 0.08) lactate and plasma urea nitrogen (*P* = 0.07) concentration associated with SBC addition. Fat-corrected milk yield (FCM_4%_) and composition was not affected (*P* > 0.27) by SBC; however, there was a tendency for decreased total milk solids (*P* = 0.07) and milk fat (*P* = 0.08) associated with 23% SBC treatment. There was no treatment × technique interaction (*P* > 0.05) effect on gas measurements. A maximum reduction (*P* = 0.01) in CH_4_ yield (g/kg DM) and intensity (g/kg milk) of 11 and 20%, respectively, was observed for the 14% SBC inclusion. Compared to the week of mask measurements, chambers decreased (*P* = 0.01) intake (kg/d, %BW) and increased (*P* = 0.05) FCM_4%_. The face-mask method over estimated O_2_ consumption by 5%. The face-mask method accurately predicted daily CH_4_ emissions when compared to the chamber at the same time-point. However, there was a linear bias of CH_4_ outputs so further evaluation of the calculation of total CH_4_ from a spot measurement is required.

## Introduction

The expansion of the biofuel industry has contributed, in part, toward a rise in livestock grain and oilseed prices. As a result, the utilization of co-products derived from biofuel manufacturing is of increasing popularity as a low cost feed alternative (1). Due to the high fat content of biofuel co-products (2–4), there is a potential for the utilization of these feedstuffs as a methane (CH_4_) mitigation tool. Dietary fats have been proven to decrease CH_4_ emissions through the suppression of methanogen and ciliate protozoa populations, dilution through replacement of fermentable carbohydrates, reduction of ruminal organic matter fermentation and biohydrogenation of free unsaturated fatty acids (5–7). Dried distillers grain originating from bioethanol production have been proven to decrease enteric CH_4_ emissions (4, 8), however, more research efforts are needed to investigate the mitigating properties of biodiesel co-products. As the desire for the use of all biofuel co-products in livestock production increases, an investigation into biodiesel co-products as a mitigation strategy is of significance to livestock industries worldwide.

Soybeans are one of the most common feedstock for biodiesel manufacturing globally (9). The resultant high fat co-product, referred to as soybean cake (SBC), obtained by a physic process (pressure and heat) of oil extraction, is an available co-product and possibly an alternative to soybean meal (SBM). The effective replacement of SBM with SBC in livestock diets, in addition to the potential mitigating properties of this feed source, has wide-reaching economic and environmental importance. Additionally, SBC may be an option for the organic dairy market, which requires milk production to be free of chemicals.

A need for inexpensive and convenient methods in the estimation of livestock CH_4_ emissions has been identified. Currently, the respiration chamber is considered the gold standard method for quantification of enteric CH_4_ emissions (10). However, chamber use is limited on a practical research basis and cannot be implemented on farm due to costly infrastructure, lengthy observation periods, and alterations to animal behavior (10–12).

The investigation of alternative CH_4_ measurement techniques in recent studies has focused primarily on the calculation of daily emissions from spot samples of eructated and respired air. Garnworthy et al. (11) and Huhtanen et al. (10) accurately predicted daily CH_4_ emissions based on samples obtained via specialized feeders. Based on these outcomes, and utilizing existing chamber infrastructure, an updated face-mask method involving the measurement of respired and eructated air has been proposed as a more practical means by which total daily emissions can be quantified (13). Previous use of the face-mask technique involved 30 min measurement periods taken every 2–3 h throughout the day (14). The high frequency of measurements were found to lead to altered animal behavior and therefore did not represent actual emissions. Due to the strong correlation between total daily emissions and spot samples at specific times post feeding, the number of samples taken throughout the day could be reduced, minimizing the impact on animal behavior.

The objective of this study was to (a) evaluate the effect of replacing soybean meal (SBM) with soybean cake (SBC) on feeding behavior, rumen fermentation, milk production, nutrient digestibility, and CH_4_ emissions and (b) assess whether the proposed face-mask method could accurately predict daily CH_4_ emissions when compared to the gold standard respiration chamber. It was hypothesized that the SBC would reduce CH_4_ emissions in crossbred dairy cows, without impacting animal intake or performance and that the face-mask method could accurately predict daily CH_4_ production.

## Materials and Methods

The experiment was conducted in the Bioenergetics Laboratory of the Brazilian Agricultural Research Corporation (EMBRAPA), at the Multi-use Complex on Livestock Bioefficiency and Sustainability at Embrapa Dairy Cattle, in Coronel Pacheco, Minas Gerais, Brazil. All animal care and handling procedures were approved by the Embrapa Dairy Cattle Animal Care and Use Committee (Juiz de Fora, Minas Gerais, Brazil; Protocol No. 28/2014).

### Animals, Experimental Design, and Treatments

Thirty-two lactating crossbred cows (5/8 Holstein × Gyr) were selected based on days in milk (DIM; 112 ± 25.1), milk yield (20.8 ± 2.92 kg/d), and body weight (BW; 551 ± 45.4). Animals were randomly assigned to four dietary treatments (*n* = 8 per treatment) over 75 days. The first 21 days consisted of a dietary adaptation, followed by 54 days measurement period. All cows were exposed to both the chamber and face-mask across the final 28 days of the measurement period. The dietary treatments consisted of (1) 0% SBC (control diet; CON), (2) 6% SBC (6% SBC), (3) 14% SBC (14% SBC), and (4) 23% SBC (23% SBC) on a DM basis. Cows were housed in a covered freestall for the experimental period, except during milking, and periods of methane measurement using the respiration chambers and face-mask. Respiration chambers and the face-mask technique were conducted within a controlled environment facility.

### Feed Sampling and Calculation of DM Intake

Cows were fed once daily at 10:00–11:00 for *ad libitum* intake (5–10% orts). Diets were formulated using the Large Ruminant Nutrition System (LRNS; version 1.0.29) to meet the protein and energy requirements of a 550 kg cow producing 25 kg/d of milk (3.9% fat; 3.0% true protein) and consuming 18.5 kg DM/d, as according to the NRC (15). Due to protein content variation between the SBM and SBC, the four treatment diets were formulated to be iso-proteic, hence treatment diets differed in concentrate DM content (Table 1).

**Table 1:** Ingredients and chemical composition of diets.

	**Treatment**			
**Item**	**Control**	**6% SBC**	**14% SBC**	**23% SBC**
**Ingredients (% DM)**				
Corn silage	49.2	49.2	49.2	49.2
Tifton hay	5.4	5.4	5.4	5.4
Corn grain, fine ground	22.2	21.9	21.6	21.3
Soybean meal^a^	22.2	16.5	8.6	0.0
Salt	0.3	0.3	0.3	0.3
Soybean cake (SBC)^b^	0.0	5.9	14.1	23.0
Limestone	0.7	0.7	0.7	0.7
Mineral supplement	0.2	0.2	0.2	0.2
**Chemical composition**				
Dry matter (%)	43.3	43.3	43.2	43.2
Crude protein (CP, % DM)	17.3	17.3	17.3	17.3
Neutral detergent fiber (NDF, % DM)	29.4	27.9	29.2	28.9
NFC^c^ (% DM)	44.5	46.4	43.9	43.1
Ether extract (EE, % DM)	3.1	3.6	4.2	4.9
Organic matter (% DM)	94.3	95.2	94.6	94.2

*Control, no SBC; 6% SBC, 6% DM of soybean cake replacing soybean meal; 14% SBC, 14% DM of soybean cake replacing soybean meal; 23% SBC, 23% DM of soybean cake replacing soybean meal*.

a *Nutrient content of soybean meal composition (% in the DM): 48.8 CP, 14.6 NDF, 28.3 NFC, 1.7 EE, 6.6 ash*.

b *Nutrient composition of soybean cake: 44.1 CP, 9.2 NDF, 29.1 NFC, 10.2 EE, and 7.5 ash*.

c *NFC = non-fibrous carbohydrates = 100 – (CP + NDF + EE + ash)*.

The freestall was fitted with 32 electronic feed bins and head gates (AF-1000-MG, Intergado Ltd., Contagem, Minas Gerais, Brazil), as well as six electronic water troughs. Feed and water troughs were attached to weight measurement platforms (WD-1000, Intergado Ltd., Contagem, Minas Gerais, Brazil) and radio frequency identification (RFID) antennas that monitored individual feed and water intake, as well as feeding and drinking behavior (16). Cows were fitted with an ear tag containing a unique passive transponder (FDX–ISO 11784/11785; Allflex, Joinville, SC, Brazil) in the right ear, and each feed bin was randomly assigned to a single cow. Each calorimetric chamber was also fitted with the Intergado feed technology and water trough. Chamber DMI was measured during the 2 days in which the cows were in the chamber. Face-mask DMI was considered to be the intake of the cow in the free stall for the 2 days in which the animal underwent face-mask measurement.

Feed bin construction, dimensions and operation are as described by Chizzotti et al. (16). The visit duration, the number of visits to feed and water troughs, and fresh feed and water intake data were exported from Intergado web software for report generation. Body weight was also recorded each time cows consumed water via the platform with load cells associated with the water bins and exported from Intergado web software.

Dietary forage, concentrate mix, and orts were sampled weekly for DM determination. Diet intake for each cow was calculated using the Intergado system output and laboratory DM of diets.

### Collection of Rumen Samples

Rumen samples (60 mL/cow) were collected on the last week of the experimental trial, 4 h after feeding via a stomach tube (17, 18). Samples were preserved for volatile fatty acids (VFA) analysis by adding 1 mL of 20% (w/vol) metaphosphoric acid to 5 mL of sample (1:5 dilution). Samples were frozen at −20°C until analysis.

### Collection of Blood Samples

During the first 7 days of the measurement period, blood samples were collected from the coccygeal vein, 2 and 6 h after feeding. Blood was collected in 4 mL vacutainer tubes (BD vacutainer systems, Plymouth, UK) and serum was extracted following centrifugation at 1,800 × *g* for 20 min at 4°C. Samples were stored at −20°C until analysis.

### Milk Yield and Collection of Milk Samples

Cows were milked twice daily at 06:00 and 14:30 h. Milk yield was automatically recorded for each cow at each milking (DeLaval Alpro MM27BC milk meter system; DeLaval International, Tumba, Sweden). Composite milk samples (100 mL) were collected weekly at both a.m. and p.m. milkings for 3 consecutive days during each week of the measurement period, pooled and then analyzed for fat, protein, lactose, and urea-N content. These samples were preserved with Bronopol^®^ (0.5 g/100 mL of milk) and stored at 4°C until analysis. An additional set of milk samples were collected separately at a.m. and p.m. milkings in 15-mL flasks containing no preservatives. These samples were immediately frozen at −20°C until analyzed for fatty acid composition.

Milk composition was corrected for volume differences between the a.m. and p.m. milking. Total milk production was corrected by adjusting the fat content to 4% (FCM_4%_; fat-corrected milk) by the equation (15):

$$\begin{array}{l} \text{FC}{\text{M}}_{4\text{\%}}=\left(0.4\times \text{kg}/\text{d milk}\right)\\ \;+\left[15\times \frac{\left(\text{fat production}\times \text{milk yield}\right)\;}{100}/7\right] \end{array}$$

### Collection of Fecal and Urine Samples

Fecal samples were taken twice daily for 5 days at 09:00 and 14:00 directly from the rectum and frozen at −20°C. Acid-insoluble ash was used as internal marker for total-tract digestibility estimation. Fecal samples were analyzed for DM, OM, N, NDF, and ether extract content. Urine samples were collected once a day, across 3 consecutive days. A subsample of pooled urine was acidified with H_2_SO_4_ to evaluate creatinine concentrations (19) and urine N content (AOAC, method 954.01). Daily urine volume (DUV, kg/d) was estimated by metabolic weight (BW^0.75^) and urinary creatinine concentrations according to the equation proposed by Valadares et al. (20):

$$\text{DUV}=\frac{{\text{BW}}^{\text{0}.\text{75}}\times \text{29}}{\text{creatinine concentration }(\text{mg/L})}$$

### CH_4_ Measurement in Respiration Chambers

During the last weeks of measurements cows were randomly selected (one animal per treatment per chamber per collection period) and moved to an open circuit respiration chamber for 2 × 20 to 22 h periods for determination of CH_4_ and CO_2_ production, and O_2_ consumption. All cows were trained and pre-conditioned for 2 weeks to the chamber and face-mask technique prior to the onset of the experiment. The four chambers were equipped as described by Machado et al. (21). Chambers were built in a pair wise manner with one cow housed per chamber. Days in which cows entered the chambers were staggered across 4 weeks, as there were only four chambers available at onetime. Each cow entered the chamber after the a.m. milking (06:00) and was removed for 2 h during the measurement period for p.m. milking (14:30). The chamber doors remained open during this time for cleaning and provision of fresh feed, twice a day from here on forward. Upon re-entry to the chamber, conditions were assumed to stabilize after 30 min. The chambers were maintained under thermal neutral conditions for crossbred Holstein × Gyr cows (Temperature: 23 ± 1°C; Relative humidity: 65 ± 5%). The animals were weighed before and after entering the chamber. The gas exchanges (O_2_ input, CO_2_ and CH_4_ output) were calculated according to Machado et al. (21). Heat production (HP, Kcal/day) was calculated as according to Brouwer (22):

$$\begin{array}{l} \text{HP}\left(\text{Kcal}/\text{d}\right)=\left(3.866\times \text{V}{\text{O}}_{2}\right)+\left(1.200\times \text{VC}{\text{O}}_{2}\right)\\ \;-\left(0.518\times \text{VC}{\text{H}}_{4}\right)-\left(1.431\times \text{UN}\right) \end{array}$$

Where: VO_2_ = volume of oxygen (L/d); VCO_2_ = volume of carbon dioxide (L/d); VCH_4_ = volume of methane (L/d); UN = total urine nitrogen (g/d).

### Methane Estimation Using a Face-Mask Technique

**Figure 1:**
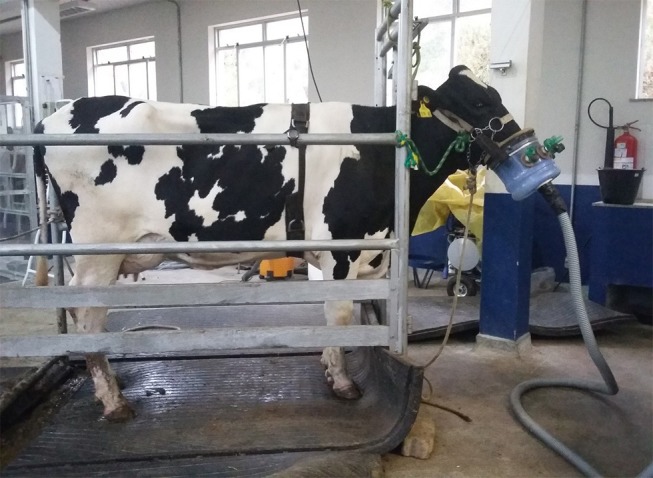
Galvanized iron frame for animal restraint, and face-mask construction and application for the estimation of daily CH_4_ methane production in cattle. Cow is also fitted with a heart rate monitor.

For spot measurements of CH_4_, CO_2_, and O_2_ exchange, a face-mask method was employed. The mask was built using a 8 L polyethylene water container fitted with uni-directional valves (Figure 1) which prevented the rebreathing of exhaled air and allowed the external air to enter the mask (Era Mask, 220 mm × 160 mm × 77 mm, Biomedtech, Victoria, Australia). One cow at a time was taken to the controlled environment facility and placed within a squeeze chute for face-mask measurements. Through an inflatable circular rubber tube positioned around the animal's muzzle, the mask remained inflated and was positioned by a nylon strap attached around the neck of the cow (Figure 1). Gas sampling was performed by a tube that connected the mask to the flux meter and then gas analyzers, and sampling was performed at a rate of 0.3 L/min for each kg of live weight [Sable International Systems, Las Vegas, USA; (13)]. The calibration procedures of the system, sampling and data analysis were as described by Oss et al. (13). Briefly, the 30 min period consisted of a 5 min baseline, 20 min mask exhaust measurement, and another 5 min baseline. Measurement using the face-mask was conducted on 2 consecutive days, 4 h post feeding to avoid sampling at peak CH_4_ production soon after feeding. This prevented the over estimation of daily CH_4_ emissions of each cow. The CH_4_ emissions data was recorded with the Sable System (Sable Systems International, Las Vegas, NV, USA) attached to the face-mask. Air flow rate (100 L/min) through the mask was controlled and measured by a mass flow controller (Flow Kit 500H; Sable Systems International, Las Vegas, NV, USA). Gas samples from the face-mask and ambient air were continuously sampled through Bev-A-Line tubes at 1 min intervals. The CH_4_ and CO_2_ analyzers were calibrated daily using N gas to zero the analyzers. Methane production (VCH_4_; mL/min) was calculated as according to Oss et al. (13);

$$\begin{array}{l} VCH_{4}=\left[STDfr\times \left(CH_{4}fm-CH_{4}b\right)\right] \end{array}$$

Where STD = standard deviation of the flow rate; CH_4_fm = CH_4_ measured from face-mask; CH_4_b = CH_4_ measured from baseline).

Daily CH_4_ was calculated by multiplying VCH_4_ by 1.44 to convert to L/d and then converted to g/d (1 g CH_4_ = 1.4 L CH_4_). Cows were individually restrained in a galvanized iron frame measuring 202 × 81 × 171 cm (length × width × height; Figure 1) for the duration of the measurement period.

### Chemical Analyses

The DM content of feed and fecal samples were determined by oven-drying at 55°C for 72 h. Dried samples were ground through a 1-mm screen and analyzed for neutral detergent fiber (NDF) as described by Van Soest et al. (23), modified for an Ankom 200/220 Fiber Analyzer (Ankom Technol. Corp., Fairport, NY, USA). Amylase was used but sodium sulfite was not included in the NDF analyses and is expressed inclusive of residual ash. Ash content was determined after 2 h of oxidation at 600°C in a muffle furnace (24) (method 942.05). Nitrogen was quantified by Kjeldahl method (method 984.13). Ether extract (EE) was determined by extraction with ether as described for lipid extraction [method 920.39; (24)]. Non-fibrous carbohydrate (NFC) was calculated as by Mertens (25):

$$\begin{array}{l} \text{NFC}=100-\left(\text{CP}+\text{NDF}+\text{EE}+\text{Ash}\right)\; \end{array}$$

The VFA concentrations were analyzed using high-performance liquid chromatography (Dionex Ultimate 3000 Dual Detector HPLC—Dionex Corporation, Sunnyvale, CA, USA [Phenomenex Rezex ROA ion exchange column, 300 × 7.8 mm]). Blood samples were analyzed for non-esterified fatty acids (NEFA; Randox, County Antrim, UK), β-Hydroxybutyric acid (BHBA; RANBUT assay, Randox, County Antrim, UK), cholesterol, triglycerides and urea-N (Randox, County Antrim, UK) using a microplate spectrophotometer (Eon, BioTek, Winooski, USA).

A Bentley Fourier-Transform Infrared Spectrometer System (Bentley FTS, Bentley Instruments, Chaska, MN) was used to determine milk fat, protein, and lactose. A commercial calorimetry kit (Sigma Diagnostics, St. Louis, MO) was used to analyze milk samples for urea-N. Milk FA composition was determined as described elsewhere (26). The Kjedahl method (AOAC, method 954.01) was used to determine fecal N content and total fecal production was estimated by the insoluble ash technique (27).

### Statistical Analysis

The raw intake data obtained from the Intergado system were used to calculate total daily feed and water intake, the number of feed and water bin visits with and without consumption, and the duration of feed and water bin visits.

Data were analyzed with cow as the experiment unit for all variables. Feeding behavior, rumen fermentation parameters, and digestibility data were analyzed as a completely randomized design using the mixed procedure of SAS (28) with treatment as a fixed effect and cow nested within group as a random effect. For CH_4_ measurement data, treatment, technique, and the interaction of treatment × technique were included as fixed terms and day of measurement was treated as a repeated measure. Time of sampling was treated as a repeated measure for blood variables. Since milk samples were collected separately at a.m. and p.m. milkings for fatty acid analysis, treatment, milking time and interaction were used as fixed term. Minimum values of Akaike's Information Criterion were used to choose covariance structure for each repeated measures analysis. Orthogonal polynomial contrasts were used to determine linear (L) and quadratic (Q) responses of SBC (0, 6, 14, and 23% of SBC) replacing SBM. Significance was declared if *P* ≤ 0.05 and tendencies 0.05 < *P* ≤ 0.10.

The validity of the face-mask method to measure CH_4_ was evaluated by regressing residuals (respiration chamber—face-mask) as a function of face-mask centered predicted values (29, 30) using the REG procedure (SAS, 9.4). Regressions with both intercept (mean bias) and slope (slope bias) not different from zero (*P* > 0.05) were classified as potential accurate predicting equations.

## Results

### Animal Performance

There was no effect (*P* ≥ 0.21) of replacing SBM with SBC on dry matter intake (DMI), feeding behavior, water consumption or BW (Table 2). Replacing SBM with SBC also had no effect on total VFA production (*P* = 0.79) or the concentration of acetate, propionate and butyrate (*P* ≥ 0.27). There was a tendency (*P* = 0.08) for lactate to decrease when 6% of SBC replaced SBM in the diet. Blood metabolites including BHBA, NEFA, Triglycerides, and cholesterol were not affected (*P* ≥ 0.17) by any treatment (Table 3). Serum concentrations of BHBA, NEFA, triglycerides, or cholesterol did not present an isolated treatment or collection time effect (*P* ≥ 0.67). There was interaction effect (*P* ≤ 0.03) of treatment × hour for NEFA and cholesterol. The 14% SBC and 23% SBC treatments resulted in a lower (*P* < 0.01) serum cholesterol concentration at 6 h post-feeding compared to 2 h post-feeding, while NEFA concentration for cows fed the control diet was greater (*P* < 0.01) at 6 h post-feeding only. The urea levels differed in relation to the time of collection (*P* < 0.01) and presented a reduction trend (*P* = 0.07) with the increase of SBC in the diet.

**Table 2:** Effect of replacing soybean meal (SBM) with soybean cake (SBC) on dry matter intake (DMI), feeding behavior, water consumption, and body weight (BW) in dairy cattle.

	**Treatments**				**SEM**	***P-*value**
	**Control**	**6% SBC**	**14%SBC**	**23% SBC**		
BW (kg)	614.4	578.4	577.3	613.5	17.95	0.28
**Feeding behavior**						
DMI (kg/d)	17.4	15.8	16.1	15.9	1.13	0.75
Total visits to feed bin	41.4	28.7	40.8	41.6	7.11	0.52
Number of feed bins visited	2.4	2.2	2.8	3.2	0.49	0.45
Visits with consumption	34.1	22.5	29.1	25.6	4.65	0.35
Visits without consumption	7.3	6.4	11.9	16.0	4.68	0.45
Total time spent at feed bin (h)	2.6	2.5	2.7	2.3	0.23	0.73
Total time spent eating (h)	2.6	2.5	2.6	2.3	0.23	0.70
**Drinking behavior**						
Water intake (L/d)	59.0	60.0	59.1	59.8	3.52	0.99
Total visits to trough	4.4	4.2	5.1	3.9	0.41	0.21
Visits with consumption	4.3	4.2	5.0	3.8	0.40	0.22
Visits without consumption	0.12	0.04	0.13	0.13	0.05	0.50
Total time spent at trough (h)	0.43	0.40	0.61	0.45	0.10	0.47
Total time spent drinking (h)	0.42	0.40	0.60	0.44	0.10	0.45

*Control, no SBC; 6% SBC, 6% DM of soybean cake replacing soybean meal; 14% SBC, 14% DM of soybean cake replacing soybean meal; 23% SBC, 23% DM of soybean cake replacing soybean meal*.

**Table 3:** Effect of replacing soybean meal (SBM) with soybean cake (SBC) on volatile fatty acids (VFA) and blood metabolites in dairy cattle.

**Item**	**Treatment**				**SEM**	***P*-value**
	**Control**	**6% SBC**	**14% SBC**	**23% SBC**		
Total VFA (mM)	72.2	75.5	68.5	69.7	5.30	0.79
**VFA, mol/100 mol**						
Acetate (A)	63.2	61.7	62.6	63.1	0.68	0.37
Propionate (P)	22.7	23.3	22.0	21.9	0.62	0.36
Butyrate	14.0	15.1	15.4	15.0	0.52	0.27
Lactate	7.5	4.1	6.9	7.3	1.04	0.08
A:P ratio	2.8	2.7	2.9	2.9	0.09	0.39
**Blood metabolites**						
BHBA (mmol/L)	1.1	1.0	1.1	1.0	0.07	0.68
NEFA (mmol/L)	0.2	0.2	0.2	0.2	0.01	0.45
Triglycerides (mg/dL)	10.4	11.1	10.5	11.6	0.51	0.29
Cholesterol (mg/dL)	178.3	152.1	134.6	173.9	15.37	0.17
PUN (mg/dL)	51.0	46.3	41.8	43.7	2.59	0.07

*Control, no SBC; 6% SBC, 6% DM of soybean cake replacing soybean meal; 14% SBC, 14% DM of soybean cake replacing soybean meal; 23% SBC, 23% DM of soybean cake replacing soybean meal*.

*BHBA, β-hydroxybutrate; NEFA, non-esterified fatty acids; PUN, plasma urea nitrogen*.

*Treatment × Time effect NEFA, P = 0.03; Cholesterol Treatment × Time effect, P = 0.001*.

Replacing SBM with SBC had no effect on total daily FCM_4%_ (*P* = 0.29; Table 4). Milk protein, lactose, and urea concentrations were unaffected (P ≥ 0.27) by treatments; however, there was a trend (*P* = 0.09) toward reduced total milk solids and fat percentage as SBC increased.

**Table 4:** Effect of replacing soybean meal (SBM) with soybean cake (SBC) on milk production, milk composition, and milk fatty acids of dairy cows.

	**Treatment**				**SEM**	***P*-value**
	**Control**	**6% SBC**	**14% SBC**	**23% SBC**		
Fat corrected milk yield (FCM_4%_, kg/d)	19.4	21.5	21.3	19.4	1.03	0.29
**Milk composition (%)**						
Total milk solids	12.4	12.6	12.2	11.6	0.27	0.07
Fat	4.6	4.8	4.4	4	0.23	0.09
Protein	3.4	3.3	3.3	3.3	0.07	0.77
Lactose	4.4	4.4	4.5	4.3	0.07	0.51
Urea	19.3	17.7	17	16.1	1.16	0.27
**Fatty acid (FA)**						
C4:0	3.56	3.55	3.54	3.46	0.119	0.91
C5:0	0.04^a^	0.03^ab^	0.03^b^	0.02^b^	0.004	0.01
C6:0	2.25	2.18	2.23	2.07	0.085	0.44
C7:0	0.04^a^	0.03^ab^	0.03^b^	0.02^b^	0.004	0.02
C8:0	1.35	1.28	1.36	1.19	0.067	0.23
C9:0	0.05	0.04	0.04	0.03	0.006	0.18
C10:0	2.96	2.67	3	2.58	0.199	0.32
C10:1 *c*-9	0.35	0.3	0.29	0.28	0.024	0.13
C11:0	0.08	0.07	0.08	0.08	0.008	0.55
C12:0	3.66	3.11	3.42	2.91	0.237	0.12
C12:1 *c*-9 + C13:0	0.25	0.2	0.21	0.19	0.021	0.21
C14:0 *iso*	0.11^a^	0.10^ab^	0.08^c^	0.09^bc^	0.007	0.01
C14:0	10.4	9.75	10.3	9.32	0.348	0.11
C15:0 *iso*	0.23^a^	0.22^a^	0.18^c^	0.17^b^	0.006	<.001
C15:0 *anteiso*	0.42	0.42	0.42	0.38	0.017	0.25
C14:1 *c*-9	1.33	1.05	0.97	1.15	0.103	0.07
C15:0	1.05	1	0.95	0.88	0.06	0.21
C16:0 *iso*	0.25^ab^	0.25^a^	0.20^c^	0.21^bc^	0.016	0.03
C16:0	29.6^a^	27.6^ab^	25.5^bc^	24.9^c^	0.819	<.001
C16:1 t-9 + C17:0 *iso*	0.33	0.35	0.29	0.31	0.016	0.05
C16:1 *t*-12	0.15	0.14	0.15	0.14	0.008	0.72
C16:1 *c*-9 + C17:0 *anteiso*	2.04^a^	1.77^ab^	1.53^b^	1.67^b^	0.125	0.03
C17:0	0.46^a^	0.50^a^	0.47^a^	0.42^b^	0.015	0.01
C18:0 *iso*	0.06^a^	0.05^b^	0.04^b^	0.04^b^	0.003	<0.01
C17: 1 *c*-9	0.19	0.19	0.17	0.16	0.014	0.34
C18:0	9.0^b^	11.4^a^	11.5^a^	11.4^a^	0.58	0.01
C18:1 *t*-4	0.04	0.04	0.03	0.04	0.003	0.33
C18:1 *t*-5	0.03	0.03	0.03	0.02	0.003	0.19
C18:1 *t*-6, *t*-7, *t*-8	0.23^c^	0.28^bc^	0.33^ab^	0.39^a^	0.022	<0.01
C18:1 *t*-9	0.23^b^	0.26^b^	0.33^a^	0.33^a^	0.014	<.001
C18:1 *t*-10	0.28^c^	0.38^bc^	0.56^ab^	0.76^a^	0.092	<0.01
C18:1 *t*-11	0.86^b^	1.05^b^	1.43^a^	1.48^a^	0.111	<0.01
C18:1 *t*-12	0.29d	0.38^c^	0.49^b^	0.55^a^	0.023	<0.01
C18:1 *t*-13, *t*-14	0.40^b^	0.42^b^	0.48^ab^	0.51^a^	0.032	0.01
C18:1 *c*-9	19.0^b^	20.9^ab^	20.8^ab^	22.8^a^	0.789	0.01
C18:1 *c*-11	0.60^b^	0.73^a^	0.72^a^	0.73^a^	0.027	<0.01
C18:1 *c*-12	0.28^c^	0.36^b^	0.45^a^	0.48^a^	0.026	<0.01
C18:1 *c*-13	0.09	0.07	0.07	0.08	0.005	0.09
C18:1 *t*-16	0.24^d^	0.27^c^	0.32^b^	0.36^a^	0.011	<0.01
C19:0 + C18:1 *c*-15	0.08^a^	0.07^b^	0.08^ab^	0.09^a^	0.004	0.08
C18:2 *t*-9, *t*-12	0.03^a^	0.02^b^	0.02^c^	0.02^bc^	0.002	<0.01
C18:2 *c*-9, *t*-12	0.06^a^	0.05^b^	0.05^b^	0.07^a^	0.004	0.01
C18:2 *t*-9, *c*-12	0.05^a^	0.03^b^	0.02^c^	0.02^bc^	0.002	<0.01
C18:2 *n*-6	1.89^b^	2.08^b^	2.38^a^	2.52^a^	0.112	<0.01
C20:0	0.15	0.16	0.15	0.14	0.008	0.45
C18:3 *n*-6	0.05^a^	0.04^ab^	0.03^bc^	0.03^c^	0.003	<0.01
C18:3 *n*-3	0.31	0.35	0.36	0.37	0.016	0.06
C20:1 *c*-11	0.05	0.05	0.06	0.05	0.003	0.5
CLA *c*-9, *t*-11	0.49^b^	0.54^b^	0.72^a^	0.84^a^	0.048	<0.01
CLA *t*-9, *c*-11	0.04^a^	0.03^b^	0.03^b^	0.03^ab^	0.003	0.03
CLA *t*-10, *c*-12	0.03^a^	0.02^a^	0.01^b^	0.01^b^	0.003	<0.01
C21:0	0.04^a^	0.03^b^	0.02^bc^	0.02^c^	0.002	<0.01
C20:2 *n*-6	0.04^a^	0.03^b^	0.02^b^	0.02^b^	0.003	<0.01
C22:0	0.15^a^	0.10^b^	0.08^c^	0.08^c^	0.008	<0.01
C20:3 *n*-6	0.06	0.07	0.06	0.06	0.005	0.08
C20:4 *n*-6	0.18^a^	0.13^b^	0.1^c^	0.09^c^	0.011	<0.01
C23:0	0.05^a^	0.04^b^	0.03^c^	0.03^c^	0.004	<0.01
C20:5 *n*-3	0.06^a^	0.04^b^	0.03^c^	0.03^c^	0.003	<0.01
C24:0	0.05^a^	0.05^a^	0.03^a^	0.03^a^	0.005	<0.01
C22:5 *n*-3	0.06^ab^	0.07^a^	0.06^bc^	0.05^c^	0.003	0.01
Total SFA	66.1^a^	64.6^a^	63.7^a^	60.4^b^	1.058	<0.01
Total MUFA	24.6^c^	26.9^bc^	27.8^ab^	30.4^a^	0.921	<0.01
Total PUFA	3.36^c^	3.53^bc^	3.90^ab^	4.12^a^	0.175	0.01
*Σ n*-3FA	0.43	0.46	0.44	0.45	0.018	0.98
*Σ n*-6 FA	2.22^b^	2.37^ab^	2.61^a^	2.70^a^	0.122	0.03
*n*-6:*n*-3 FA ratio	5.24^b^	5.17^b^	6.0^a^	6.08^a^	0.195	<0.01

*Control, no SBC; 6% SBC, 6% DM of soybean cake replacing soybean meal; 14% SBC, 14% DM of soybean cake replacing soybean meal; 23% SBC, 23% DM of soybean cake replacing soybean meal*.

*Means followed by a lowercase superscript are significant different at P < 0.05*.

*SFA, saturated fatty acids; MUFA, monounsaturated fatty acids; PUFA, polyunsaturated fatty acids*.

The replacement of SBC with SBM decreased (*P* ≤ 0.05) the contents of *iso* C14:0, *iso* C15:0, *iso* C16:0, *iso* C18:0, C16:0, *trans*-10, *cis*-12 CLA, C21:0, and total SFA in milk fat, but increased (*P* ≤ 0.05) the contents of C18:0, *cis*-9 C18:1, most *trans*-C18:1 isomers (except *trans*-4 and *trans*-5 C18:1), *cis*-11 C18:1, *cis*-12 C18:1, C18:2 n-6, *cis*-9, *trans*-11 CLA, total MUFA, total PUFA, and the n-6:n-3 FA ratio (Table 4).There was no effect of replacing SBM with SBC on apparent nutrient digestibility (Table 5).

**Table 5:** Effect of replacing soybean meal (SBM) with soybean cake (SBC) on apparently nutrient digestibility in dairy cows.

	**Treatment**				**SEM**	***P*-value**
	**Control**	**6% SBC**	**14% SBC**	**23% SBC**		
**Apparent digestibility, %**						
Dry matter	81.9	81.7	81.6	81.9	0.70	0.98
Organic matter	82.6	82.6	82.4	82.6	0.73	0.99
Crude protein	83.9	83.0	81.9	82.7	0.72	0.24
Non-fiber carbohydrates	91.6	93.7	92.4	92.5	0.70	0.17
Neutral detergent fiber	67.9	65.2	66.9	66.9	1.24	0.50
Ether extract	85.8	85.7	85.3	86.9	1.52	0.89

*Control, no SBC; 6% SBC, 6% DM of soybean cake replacing soybean meal; 14% SBC, 14% DM of soybean cake replacing soybean meal; 23% SBC, 23% DM of soybean cake replacing soybean meal*.

### Methane Measurements and Techniques

The replacement of SBM by SBC had no effect (*P* > 0.23) on DMI, FCM_4_% yield (kg/d), CH_4_ production (g/d, g/kg BW^0.75^), CO_2_ production, O_2_ consumption or heat production. However, 14% SBC reduced (*P* ≤ 0.01) CH_4_ yield (g/kg DMI) and both 14% SBC and 23% SBC reduced CH_4−_ intensity (g/kg milk; Table 6). The chamber technique for CH_4_ measurement resulted in a decrease (*P* = 0.01) in DMI (kg/d; %BW) and an increase in FCM_4%_ (*P* = 0.05). There was no technique effect (*P* ≥ 0.24) on CH_4_ production reported as g/d or g/kg BW^0.75^, however there was an effect of technique (*P* = 0.01) on CH_4_ reported as g/kg DMI. The mask method underestimated (*P* < 0.01) CH_4_ (g/kg DMI) by 9.1% as compared to the chamber.

**Table 6:** Effect of replacing soybean meal (SBM) with soybean cake (SBC) on enteric CH_4_ and CO_2_ outputs, O_2_ consumption and heat production in dairy cows.

	**Treatment^1^**					**Technique**			***P*****-value**	
	**Control**	**6% SBC**	**14% SBC**	**23% SBC**	**SEM**	**Chamb**	**Mask**	**SEM**	**Treat**	**Tech**
DMI (kg/d)	18.9	16.7	18.15	17.6	0.76	16.7^a^	17.9^b^	0.50	0.63	0.01
DMI (%BW)	2.95	3.02	3.35	3.01	0.16	3.0^a^	3.2^b^	0.10	0.30	0.01
FCM_4%_ (kg/d)	17.9	19.0	21.1	19.9	1.30	20.2^a^	18.8^b^	0.78	0.37	0.05
**CH**_**4**_										
g/day	354.2	354.0	333.8	334.5	13.54	342.6	345.7	9.96	0.54	0.80
g/kg BW^0.75^	3.0	3.11	2.95	2.81	0.104	2.97	2.98	0.077	0.24	0.92
g/kg DMI	20.7^ab^	21.3^a^	18.3^c^	19.3^bc^	0.67	20.9^a^	19.0^b^	0.50	0.01	<0.01
g/kg milk	19.6^a^	18.7^ab^	15.8^c^	17.3^bc^	0.81	17.4	18.3	0.60	0.01	0.25
**CO**_**2**_										
g/day	11401	11289	11490	11314	406.0	11267	11482	278.0	0.98	0.49
g/kg BW^0.75^	96.9	99.2	101.7	95.1	3.18	97.5	99.0	2.20	0.49	0.55
g/kg DMI	663.8	680.2	632.8	657.4	18.25	681.9^a^	635.2^b^	13.62	0.33	0.01
g/kg milk	640.3	604.1	545.4	595.4	32.43	574.8	617.8	22.24	0.23	0.08
**O**_**2**_										
g/day	7428	7362	7487	7408	21.5	7226^b^	7616^a^	168.5	0.99	0.04
g/kg BW^0.75^	63.1	64.7	66.3	62.3	1.93	62.6^b^	65.7^a^	1.33	0.46	0.04
g/kg DMI	431.4	444.7	413.3	429.8	12.39	437.8	421.8	9.24	0.37	0.17
g/kg milk	418.6	394.9	356	390.4	21.51	369.5^b^	410.5^a^	14.43	0.23	0.01
**Heat production**										
MJ/d	112.3	111.2	113.2	111.9	3.80	109.7	114.6	2.58	0.99	0.08
MJ/kg BW^0.75^	0.95	0.98	1.00	0.94	0.03	0.95	0.99	0.02	0.46	0.09

1 *Lsmeans of treatment effect (average values between chamber and face-mask techniques)*.

*Control, no SBC; 6% SBC, 6% DM of soybean cake replacing soybean meal; 14% SBC, 14% DM of soybean cake replacing soybean meal; 23% SBC, 23% DM of soybean cake replacing soybean meal*.

*Chamb, Chamber technique; Mask, Face-mask technique; DMI, dry matter intake; BW^0.75^, metabolic body weight*.

*Means followed by a lowercase superscript are significantly different at P < 0.05*.

*FCM_4%_, fat correct milk yield*.

The production of CO_2_ was not affected (*P* ≥ 0.23) by SBC inclusion, however there was a technique effect (*P* = 0.01) when CO_2_ was expressed per g/kg of DMI. The dietary treatments had no effect on O_2_ (g/d) consumption, however the mask overestimated (*P* = 0.04) O_2_ consumption (Table 6). Replacing SBM with SBC had no effect (*P* = 0.99) on heat production with only a trend (*P* = 0.08) for the technique to alter heat production.

**Figure 2:**
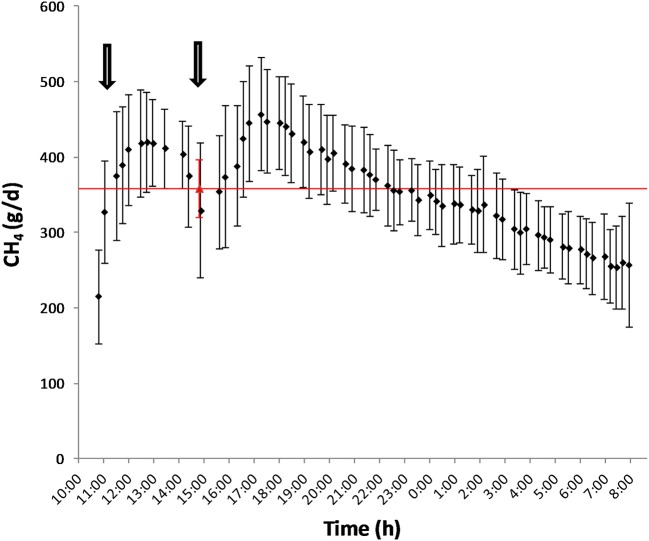
Diurnal pattern of CH_4_ production (g/d) as measured by the chamber (♦) and by the face-mask (▵) after feeding. The arrows represent the schedule of feeding (10:00 and 15:00).

**Figure 3:**
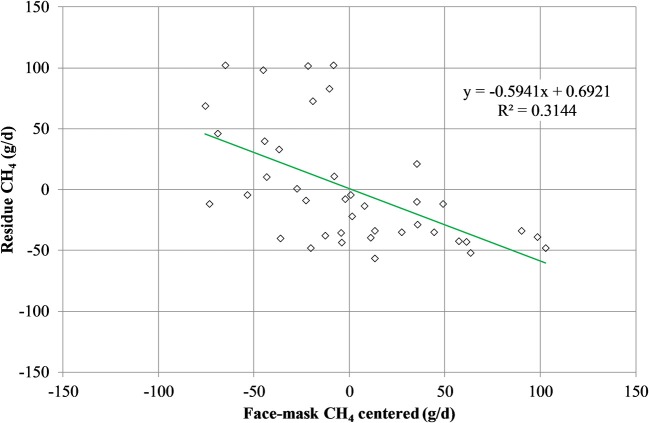
Plot of residue (respiration chamber minus face-mask) CH_4_ production (g/d) vs. centered (LSMEANS minus averaged of face-mask measurement) CH_4_ production as measured by the face mask. Absence of bias occurred when intercept is equal to 0.

Mean biases were significant for CH_4_, g/kg DMI and heat production, Kcal/BW^0.75^ (*P* < 0.01; Table 7). All measurements were significant (*P* < 0.05) for linear bias with a maximum bias of 102.8, 8.5, 5.5, 1.0, and 41.5 and a minimum bias of −75.5, −4.0, −6.9, −1.1, and −55.3 for CH_4_ (g/d), CH_4_ (g/kg DMI), CH_4_ (g/kg milk), CH_4_ (g/kg BW^0.75^), and heat production (Kcal/BW^0.75^), respectively (Table 7). The variation in CH_4_ (g/d) observed in the chamber is shown in Figure 2. Methane measured in the chamber and by the face-mask at the same time (15:00 h) are the same. Plots of the regression of residuals on centered predicted values for CH_4_ are shown in Figure 3. Absence of bias occurred when intercept is equal to 0.

**Table 7:** Evaluation of bias for CH_4_ outputs (g/d, g/kg DMI, g/kg milk, and g/kg BW^0.75^) and heat production (Kcal/BW^0.75^) measured using face-mask and respiration chambers in dairy cows fed replacing concentrations of soybean meal (SBM) with soybean cake (SBC).

	**Average CH_**4**_**	**Mean bias**	***P*-value mean bias**	**Linear bias**	***P*-value linear bias**	**Maximum bias**	**Minimum bias**
CH_4_, g/d	358.5	1.17	0.850	−0.53	<0.001	102.8	−75.5
CH_4_, g/kg DMI	20.86	0.99	0.005	−0.97	<0.001	8.5	−4.0
CH_4_, g/kg milk	17.6	−0.70	0.073	−0.26	0.048	5.5	−6.9
CH_4_, g/kg BW^0.75^	3.08	0.01	0.918	−0.72	<0.001	1.0	−1.1
Heat production, Kcal/BW^0.75^	231.4	−9.41	<0.001	−0.66	<0.001	41.5	−55.3

*DMI, dry matter intake; BW^0.75^, metabolic weight*.

## Discussion

The current study evaluated the potential of SBC to replace SBM in the diet of dairy cattle. Methane yield (g/kg DMI) was reduced by 11.6% when SBC replaced SBM at 14%, compared to the control. The decrease in CH_4_ intensity (g/kg milk) was likely due to observed numerical increase in FCM_4%_ and decrease in CH_4_ (g/d). The 11.6% reduction in CH_4_ yield as observed in this study is comparable to results reported by Beauchemin et al. (6), who found that CH_4_ production was decreased by 16% in dairy cows when the diet was supplemented with canola meal as to increase the EE content of the diet to 3.9% (total diet DM). Brask et al. (31) examined different physical forms of rapeseed fat and found that CH_4_ intensity (L/kg total ingested OM) was 12.6% lower for rapeseed cake than rapeseed meal. Martin et al. (32) showed that including crude linseed reduced CH_4_ emissions (g/kg DMI) by 10% with a total diet fat content of 5.7% in dairy cattle. Further reductions were observed when extruded flaxseeds (26%) or linseed oil (49%) replaced crude linseed, keeping the same total diet fat content. McGinn et al. (33) found that 5% sunflower oil inclusion in a forage based diet resulted in a 17% reduction in CH_4_ (g/kg DMI) emissions. In the present study, 14% SBC had a larger reduction in CH_4_ yield than 23% SBC. This was possibly due to the numerical decrease in DMI and FCM_4%_ associated with the higher replacement concentration. The replacement of 6% SBM with SBC did not change CH_4_ emissions compared to the control, suggesting that the level of dietary fat was insufficient to inhibit methanogenesis.

The dietary treatments in this experiment were formulated to offer increasing dietary fat concentrations, so that the highest inclusion of SBC was lower than that as recommended by the National Research Council (15) of 6–7% total diet DM. This recommendation is given so that DMI or organic matter fermentation is not negatively affected by dietary fat content (6, 7, 32). As such, there was no effect of SBC inclusion on DMI in the current study. Similarly, replacement of rapeseed meal with rapeseed cake did not alter OM or NDF digestibility in lactating Holstein cows when dietary fat content was below 6.5% of total diet DM (31). This is in contrast to the findings of Jordan et al. (34) who observed a reduction in DMI in bulls fed a high forage diet that were supplemented with whole soybean. However, in that study, ether extract content was high (11.0%) which likely caused an inhibition of fiber digestion, increasing retention time, and contributing to rumen fill and decreased intake. Similarly, replacement of SBM with SBC in the present study did not alter nutrient digestibility or rumen fermentation.

The replacement of SBC with SBM decreased to varying degrees the contents of most iso fatty acids, C16:0, trans-10, cis-12 CLA, C21:0, and total SFA in milk fat, whereas an opposite effect was observed on milk fat contents of C18:0, cis-9 C18:1, most trans-C18:1 and cis-C18:1 isomers, C18:2 n-6, cis-9, trans-11 CLA, total MUFA, total PUFA, and the n-6:n-3 FA ratio. Except for the reduction in trans-10, cis-12 CLA, these effects are consistent with results from previous studies where plant oils rich in linoleic acid were added to dairy cow diets (35). The pronounced increase in milk fat C18:2 n-6 observed in cows fed the highest level of SBC (1.89 vs. 2.52 g/100 g of total FA for control and 23% SBC, respectively) indicates that part of the oil present in this feed ingredient escaped from rumen biohydrogenation, which is in accordance with several reports showing that milk fat C18:2 n-6 and C18:3 n-3 contents are increased to a larger extent when cows are fed with diets supplemented with whole or processed oilseeds when compared to plant oils (36). The gradual decrease in milk fat contents of iso C14:0 and iso C15:0 in response to dietary SBC inclusion is consistent with the concomitant reduction in methane output (expressed as g/kg of DM or g/kg of milk) observed in the present study; concentrations of these branched-chain fatty acids in milk fat were shown to be positively correlated to CH_4_ emissions in previous studies (37).

The respiration chamber is currently established as the “gold standard” for quantifying CH_4_ emissions from livestock (38), however there are disadvantages with its use. In this experiment it was found that DMI intake was 6.7% higher (*P* = 0.01) in the cows undergoing the mask technology than that of the chamber. One of the main disadvantages of using the chamber technique to quantify CH_4_ emissions is observed changes in behavior (12). Marked decreases in feed, as observed in the present study, and water intake can be observed with animals in respiration chambers, affecting CH_4_ measurements due to the direct relationship between CH_4_ emissions and these variables. Despite the difference in DMI between techniques, CH_4_ production (g/d and g/kg BW^0.75^) was not different (*P* ≥ 0.80) between the techniques. However, the face-mask technique predicted 9.1% less methane than the chamber when quantified on a g/kg of DMI basis and a g/kg of milk basis, also predicting less O_2_ consumption and CO_2_ production. In comparison, Oss et al. (13) found that using the face-mask technique resulted in 4% lower CH_4_ production (g/d) measured than that of the respiration chamber. The difference observed between these techniques may, in part, be due to flatus emissions which are not accounted for using this technique. However, this contribution is evaluated as being <2% of total CH_4_ production emitted from the cow (39).

The main explanation for the difference in accuracy of CH_4_ measurements obtained by the face-mask observed between Oss et al. (13) and the present study is the time at which face-mask measurements were taken. Due to labor restrictions, face-mask measurements were conducted 4 h post feeding compared to 6 h after feeding as done by Oss et al. (13). The time after feeding has been proposed to be highly correlated with average daily CH_4_ emissions (40, 41) and can account for the observed differences between these studies. Emission rates are known to follow momentary and diurnal patterns such that using a spot measurement as an average for daily production is not adequate for predicting CH_4_ production (42). As seen in Figure 2, the rate of average CH_4_ production for these animals followed the diurnal pattern in which CH_4_ production increased after feeding (3 h), reached peak at 8 h post-feeding and then steadily decreased there on after. As a 20 min spot sampling conducted in the present study, and by Oss et al. (13), does not cover the duration of a feeding and activity cycle, prediction equations need to be developed (42) which account for these patterns.

The proposed face-mask method is less expensive and can be conducted over a shorter time. However, the accuracy and precision of spot sampling techniques to measure CH_4_ emissions is still uncertain (42). Information provided by simple regression analysis can be ambiguous and lack sensitivity and, often, do not provide a proper interpretation of these relationships. The linear bias observed by the face-mask technique, though not observed by mixed model, reiterates the necessity of complex adjustment factor for daily emission calculations. However, the face-mask has shown to accurately measure emissions when conducted at the same time point as within the “gold standard” chamber. Inclusion of the uni-directional valves as an additional adjustment of the mask from Oss et al. (13), has improved the accuracy of measurement. The face-mask method in place of chamber measurements may alleviate some animal welfare concerns as animals are only strictly confined for 30 min, allowing normal behavior and activity for most of the day. However, further assessment on stress response to the face-mask technique is required.

Oss et al. (13) suggested that the face-mask technique presents a greater day and animal variation when compared to the SF_6_ and respiration chamber techniques. This method of measurement is highly dependent on the timing of measurements due to the diurnal patterns of the feeding cycle and CH_4_ emissions. However, these limitations may be overcome by obtaining data from a larger sample size of animals with strict timing of measurements as done in this experiment (43). The bias of the face-mask compared to the chamber could be minimized by increasing the number of animals used per treatment, as well as conducting two measurement periods per day (42). However, as previously discussed, increasing the times of measurements per day can also increase the risk of behavioral changes, decreasing the ability to accurately predict CH_4_ emissions. Due to restrictions in labor and technical staff the current study did not conduct more sampling events over the day. Statistical methods to assess the validity of the face-mask to accurately predict daily CH_4_ production can also alter the results as observed in this study. A standardized protocol for measurement and calculation of CH_4_ production will allow future implementation of the face-mask methods for determining CH_4_ production from ruminants.

## Conclusion

The replacement of SBM with 14% SBC reduced CH_4_ yield (g/kg DMI) and intensity (g/kg milk) in dairy cows, without having a negative impact on animal intake, rumen metabolism, FCM_4%_, or nutrient digestibility. This presents SBC as a feasible alternative to SBM, with the additional benefit of decreasing enteric CH_4_ production.

In the current study, the face-mask method was able to accurately predict daily CH_4_ emissions from spot sampling. However, this is confounded by the linear bias when evaluated using regression analysis. Therefore, for the face-mask to be accepted as a standard CH_4_ measurement tool, prediction equations need to be formulated which account for feeding behavior and the diurnal patterns of CH_4_ production.

## Data Availability

The datasets generated for this study are available on request to the corresponding author.

## Ethics Statement

All animal care and handling procedures were approved by the Embrapa Dairy Cattle Animal Care and Use Committee (Juiz de Fora, Minas Gerais, Brazil; Protocol No. 28/2014).

## Author Contributions

LGRP, AVC, RMM, TRT, FSM, and MMC: study design. SRS, RSR, JPS, ALF, RMM, and TRT: acquisition of data. SRS, RSR, ALF, and MASG: lab analysis. AVC: statistical analysis. SAT, TEB, AVC, LGRP, RMM, SRS, TRT, and MASG: writing the manuscript. All authors read, critically revised for intellectual contents and approved the final manuscript.

### Conflict of Interest Statement

The authors declare that the research was conducted in the absence of any commercial or financial relationships that could be construed as a potential conflict of interest.

## References

[1] Willson RM Wiesman Z Brenner A. Analyzing alternative bio-waste feedstocks for potential biodiesel production using time domain (TD)-NMR. Waste Manag. (2010) 30:1881–8. 10.1016/j.wasman.2010.03.008 20347586

[2] Rasco BA Dong FM Hashisaka AE Gazzaz SS Downey SE San Buenaventura ML Chemical composition of distillers' dried grains with solubles (DDGS) from soft white wheat, hard red wheat and corn. J Food Sci. (1987) 52:236–7. 10.1111/j.1365-2621.1987.tb14019.x

[3] Dugan MER Aldai N Kramer JKG Gibb DJ Juárez M Mcallister TA. Feeding wheat dried distillers grains with solubles improves beef trans and conjugated linoleic acid profiles. J Anim Sci. (2010) 88:1842–7. 10.2527/jas.2009-2575 20118430

[4] Hünerberg M Beauchemin KA Okine EK Holtshausen L Mcginn SM Harstad OM *In vitro* production of methane with increasing levels of corn- or wheat-based dried distillers' grains with solubles in a barley silage-based diet. Acta Agric Scand A Anim Sci. (2012) 62:289–94. 10.1080/09064702.2013.773057

[5] Eugène M Massé D Chiquette J Benchaar C Meta-analysis on the effects of lipid supplementation on methane production in lactating dairy cows. Can J Anim Sci. (2008) 88:331–7. 10.4141/CJAS07112

[6] Beauchemin KA Mcginn SM Benchaar C Holtshausen L. Crushed sunflower, flax, or canola seeds in lactating dairy cow diets: effects on methane production, rumen fermentation, and milk production. J Dairy Sci. (2009) 92:2118–27. 10.3168/jds.2008-1903 19389969

[7] Knapp JR Laur GL Vadas PA Weiss WP Tricarico JM. Invited review: enteric methane in dairy cattle production: quantifying the opportunities and impact of reducing emissions. J Dairy Sci. (2014) 97:3231–61. 10.3168/jds.2013-7234 24746124

[8] Hünerberg M Little SM Beauchemin KA Mcginn SM O'connor D Okine EK Feeding high concentrations of corn dried distillers' grains decreases methane, but increases nitrous oxide emissions from beef cattle production. Agric Syst. (2014) 127:19–27. 10.1016/j.agsy.2014.01.005

[9] Wiesman Z Segman O Yarmolinsky L Utilization of lipid co-products of the biofuel industry in livestock feed. In: Makkar HPS, editor Biofuel Co-products as Livestock Feed: Opportunities and Challenges. Rome: FAO (2012). p. 115–153.

[10] Huhtanen P Cabezas-Garcia EH Utsumi S Zimmerman S. Comparison of methods to determine methane emissions from dairy cows in farm conditions. J Dairy Sci. (2015) 98:3394–409. 10.3168/jds.2014-9118 25771050

[11] Garnsworthy PC Craigon J Hernandez-Medrano JH Saunders N. On-farm methane measurements during milking correlate with total methane production by individual dairy cows. J Dairy Sci. (2012) 95:3166–80. 10.3168/jds.2011-4605 22612952

[12] Lassey KR Livestock methane emission and its perspective in the global methane cycle. Aust J Exp Agric. (2008) 48:114–8. 10.1071/EA07220

[13] Oss DB Marcondes MI Machado FS Pereira LGR Tomich TR Ribeiro GO An evaluation of the face mask system based on short-term measurements compared with the sulfur hexafluoride (SF6) tracer, and respiration chamber techniques for measuring CH4 emissions. Anim Feed Sci Technol. (2016) 216:49–57. 10.1016/j.anifeedsci.2016.03.008

[14] Washburn LE Brody S Growth and Development With Special Reference to Domestic Animals. XLII. methane, hydrogen and carbon dioxide production in the digestive tract of ruminants in relation to respiratory exchange. Missouri Agricultural Experimental Station Research Bulletin, No. 263 (1937).

[15] National Research Council Nutrient Requirements of Dairy Cattle: Seventh Revised Edition, 2001. Washington, DC: The National Academies Press (2001).

[16] Chizzotti ML Machado FS Valente EEL Pereira LGR Campos MM Tomich TR. Technical note: validation of a system for monitoring individual feeding behavior and individual feed intake in dairy cattle. J Dairy Sci. (2015) 98:3438–42. 10.3168/jds.2014-8925 25771061

[17] Lodge-Ivey SL Browne-Silva J Horvath MB Technical note: bacterial diversity and fermentation end products in rumen fluid samples collected via oral lavage or rumen cannula. J Anim Sci. (2009) 87:2333–7. 10.2527/jas.2008-1472 19329475

[18] Henderson G Cox F Kittelmann S Miri VH Zethof M Noel SJ. Effect of DNA extraction methods and sampling techniques on the apparent structure of cow and sheep rumen microbial communities. PLoS ONE. (2013) 8:e74787. 10.1371/journal.pone.0074787 24040342 PMC3770609

[19] Broderick GA Radloff WJ. Effect of molasses supplementation on the production of lactating dairy cows fed diets based on Alfalfa and Corn Silage. J Dairy Sci. (2004) 87:2997–3009. 10.3168/jds.S0022-0302(04)73431-1 15375061

[20] Valadares RFD Broderick GA Filho SCV Clayton MK. Effect of replacing Alfalfa Silage with high moisture corn on ruminal protein synthesis estimated from excretion of total purine derivatives. J Dairy Sci. (1999) 82:2686–96. 10.3168/jds.S0022-0302(99)75525-6 10629816

[21] Machado FS Tomich TR Ferreira AL Cavalcanti LFL Campos MM Paiva CAV. Technical note: a facility for respiration measurements in cattle. J Dairy Sci. (2016) 99:4899–906. 10.3168/jds.2015-10298 27016825

[22] Brouwer E Report of sub-committee on constants and factors. In: Energy Metabolism. London: Academic Press; European Association for Animal Production (1965). p. 441–3.

[23] Van Soest PJ Robertson JB Lewis BA. Methods for dietary fiber, neutral detergent fiber, and nonstarch polysaccharides in relation to animal nutrition. J Dairy Sci. (1991) 74:3583–97. 10.3168/jds.S0022-0302(91)78551-2 1660498

[24] AOAC Official Method of Analysis of the Association of Official Analytical Chemists. Arlington, TX: AOAC (1990).

[25] Mertens DR. Gravimetric determination of amylase-treated neutral detergent fiber in feeds with refluxing in beakers or crucibles: collaborative study. J AOAC Int. (2002) 85:1217–40. 12477183

[26] Ribeiro RS Terry SA Sacramento JP Silveira SRE Bento CBP Da Silva EF. Tithonia diversifolia as a supplementary feed for dairy cows. PLoS ONE. (2016) 11:e0165751. 10.1371/journal.pone.0165751 27906983 PMC5132235

[27] Lee C Hristov AN. Short communication: evaluation of acid-insoluble ash and indigestible neutral detergent fiber as total-tract digestibility markers in dairy cows fed corn silage-based diets. J Dairy Sci. (2013) 96:5295–9. 10.3168/jds.2012-6442 23746591

[28] SAS Institute SAS/STAT User's Guide. Cary, NC: SAS Institute Inc (2018).

[29] St-Pierre NR. Reassessment of biases in predicted nitrogen flows to the duodenum by NRC 2001. J Dairy Sci. (2003) 86:344–50. 10.3168/jds.S0022-0302(03)73612-1 12613877

[30] Escobar-Bahamondes P Oba M Beauchemin KA. An evaluation of the accuracy and precision of methane prediction equations for beef cattle fed high-forage and high-grain diets. Animal. (2017) 11:68–77. 10.1017/S175173111600121X 27364619

[31] Brask M Lund P Weisbjerg MR Hellwing ALF Poulsen M Larsen MK. Methane production and digestion of different physical forms of rapeseed as fat supplements in dairy cows. J Dairy Sci. (2013) 96:2356–65. 10.3168/jds.2011-5239 23415515

[32] Martin C Rouel J Jouany JP Doreau M Chilliard Y Methane output and diet digestibility in response to feeding dairy cows crude linseed, extruded linseed, or linseed oil. J Anim Sci. (2008) 86:2642–50. 10.2527/jas.2007-0774 18469051

[33] Mcginn SM Beauchemin KA Coates T Colombatto D. Methane emissions from beef cattle: effects of monensin, sunflower oil, enzymes, yeast, and fumaric acid. J Anim Sci. (2004) 82:3346–56. 10.2527/2004.82113346x 15542482

[34] Jordan E Kenny D Hawkins M Malone R Lovett DK O'mara FP. Effect of refined soy oil or whole soybeans on intake, methane output, and performance of young bulls. J Anim Sci. (2006) 84:2418–25. 10.2527/jas.2005-354 16908646

[35] Kliem KE Shingfield KJ Manipulation of milk fatty acid composition in lactating cows: opportunities and challenges. Eur J Lipid Sci Technol. (2016) 118:1661–83. 10.1002/ejlt.201400543

[36] Glasser F Ferlay A Chilliard Y. Oilseed lipid supplements and fatty acid composition of cow milk: a meta-analysis. J Dairy Sci. (2008) 91:4687–703. 10.3168/jds.2008-0987 19038946

[37] Fievez V Colman E Castro-Montoya JM Stefanov I Vlaeminck B Milk odd- and branched-chain fatty acids as biomarkers of rumen function—an update. Anim Feed Sci Technol. (2012) 172:51–65. 10.1016/j.anifeedsci.2011.12.008

[38] Sejian V Lal R Lakritz J Ezeji T. Measurement and prediction of enteric methane emission. Int J Biometeorol. (2011) 55:1–16. 10.1007/s00484-010-0356-7 20809221

[39] Murray RM Bryant AM Leng RA. Rates of production of methane in the rumen and large intestine of sheep. Br J Nutr. (1976) 36:1–14. 10.1079/BJN19760053 949464

[40] Crompton LA Mills JAN Reynolds CK France J Fluctuations in methane emission in response to feeding pattern in lactating dairy cows. In: Proceedings of the 7th International Workshop: Modelling Nutrient Digestion and Utilization in Farm Animals. Paris (2011). p. 176–180.

[41] Grainger C Clarke T Mcginn SM Auldist MJ Beauchemin KA Hannah MC. Methane emissions from dairy cows measured using the sulfur hexafluoride (SF6) tracer and chamber techniques. J Dairy Sci. (2007) 90:2755–66. 10.3168/jds.2006-697 17517715

[42] Cottle DJ Velazco J Hegarty RS Mayer DG. Estimating daily methane production in individual cattle with irregular feed intake patterns from short-term methane emission measurements. Animal. (2015) 9:1949–57. 10.1017/S1751731115001676 26301870

[43] Patra AK. Recent advances in measurement and dietary mitigation of enteric methane emissions in ruminants. Front Vet Sci. (2016) 3:39. 10.3389/fvets.2016.00039 27243027 PMC4873495

